# Characterization of arteriosclerosis based on computer-aided measurements of intra-arterial thickness

**DOI:** 10.1117/1.JMI.11.5.057501

**Published:** 2024-10-10

**Authors:** Jin Zhou, Xiang Li, Dawit Demeke, Timothy A. Dinh, Yingbao Yang, Andrew R. Janowczyk, Jarcy Zee, Lawrence Holzman, Laura Mariani, Krishnendu Chakrabarty, Laura Barisoni, Jeffrey B. Hodgin, Kyle J. Lafata

**Affiliations:** a Duke University, Department of Electrical and Computer Engineering, Durham, North Carolina, United States; b University of Michigan, Department of Pathology, Ann Arbor, Michigan, United States; c Geneva University Hospitals, Department of Oncology, Division of Precision Oncology, Geneva, Switzerland; d Geneva University Hospitals, Department of Diagnostics, Division of Clinical Pathology, Geneva, Switzerland; e Emory University, Department of Biomedical Engineering, Atlanta, Georgia, United States; f Georgia Institute of Technology, Department of Biomedical Engineering, Atlanta, Georgia, United States; g University of Pennsylvania, Department of Biostatistics, Epidemiology, and Informatics, Philadelphia, Pennsylvania, United States; h Children’s Hospital of Philadelphia, Philadelphia, Pennsylvania, United States; i University of Pennsylvania, Department of Medicine, Renal-Electrolyte and Hypertension Division, Philadelphia, Pennsylvania, United States; j University of Michigan, Department of Internal Medicine, Division of Nephrology, Ann Arbor, Michigan, United States; k Arizona State University, School of Electrical, Computer and Energy Engineering, Tempe, Arizona, United States; l Duke University, Division of Artificial Intelligence and Computational Pathology, Department of Pathology, Durham, North Carolina, United States; m Duke University, Division of Nephrology Department of Medicine, Durham, North Carolina, United States; n Duke University, Department of Radiology, Durham, North Carolina, United States; o Duke University, Department of Radiation Oncology, Durham, North Carolina, United States

**Keywords:** digital pathology, computerized morphologic assessment, tissue characterization, kidney biopsy

## Abstract

**Purpose:**

Our purpose is to develop a computer vision approach to quantify intra-arterial thickness on digital pathology images of kidney biopsies as a computational biomarker of arteriosclerosis.

**Approach:**

The severity of the arteriosclerosis was scored (0 to 3) in 753 arteries from 33 trichrome-stained whole slide images (WSIs) of kidney biopsies, and the outer contours of the media, intima, and lumen were manually delineated by a renal pathologist. We then developed a multi-class deep learning (DL) framework for segmenting the different intra-arterial compartments (training dataset: 648 arteries from 24 WSIs; testing dataset: 105 arteries from 9 WSIs). Subsequently, we employed radial sampling and made measurements of media and intima thickness as a function of spatially encoded polar coordinates throughout the artery. Pathomic features were extracted from the measurements to collectively describe the arterial wall characteristics. The technique was first validated through numerical analysis of simulated arteries, with systematic deformations applied to study their effect on arterial thickness measurements. We then compared these computationally derived measurements with the pathologists’ grading of arteriosclerosis.

**Results:**

Numerical validation shows that our measurement technique adeptly captured the decreasing smoothness in the intima and media thickness as the deformation increases in the simulated arteries. Intra-arterial DL segmentations of media, intima, and lumen achieved Dice scores of 0.84, 0.78, and 0.86, respectively. Several significant associations were identified between arteriosclerosis grade and pathomic features using our technique (e.g., intima-media ratio average [τ=0.52, p<0.0001]) through Kendall’s tau analysis.

**Conclusions:**

We developed a computer vision approach to computationally characterize intra-arterial morphology on digital pathology images and demonstrate its feasibility as a potential computational biomarker of arteriosclerosis.

## 1 Introduction

Previous studies have shown that the severity of arterio- and arteriolosclerosis is associated with the risk of kidney disease progression across various disease etiologies.^1^^–^^3^ Unlike atherosclerosis, which is a specific form of arterial thickening or hardening caused by atheroma plaque build-up in the inner lining of large arteries such as the carotid artery, arterio- and arteriolosclerosis affects medium- and small-sized arteries and arterioles within the kidney parenchyma. Arterio- and arteriolosclerosis, characterized by fibrous thickening of the intima and hypertrophy of the media, and arteriolar hyalinosis, a lesion of eosin and periodic acid–schiff (PAS)-positive amorphous material deposited in the vessel wall, are manifestations of chronic damage of kidney arteries and arterioles.^4^^,^^5^ Thus, the quantification of chronic vascular changes present in renal biopsies is relevant to the diagnosis and prognostication of kidney failure and to informing treatment selection.^5^^–^^7^ In clinical practice, biopsies are visually inspected to semi-quantitatively assess the extent of intima and media thickening.^5^ However, this approach may be influenced by inherent cognitive and visual biases,^8^ with the precision and accuracy of these observations limited by high inter- and intra-observer variability.^9^^–^^12^

The recent advancement of digital pathology, paired with the availability of large digitized kidney biopsies and clinical datasets used in clinical research, clinical trials, and routine practice,^13^^–^^20^ offers unprecedented opportunities to accurately and reproducibly quantify histologic parameters and to test their clinical relevance. In addition, computational image analysis is widely utilized to convert whole slide images (WSIs) into mineable data, from which pathomic features can be automatically extracted and categorized to enhance pathologist’s ability to diagnose, prognosticate, and predict disease outcomes.^18^^,^^21^^–^^27^

Current approaches to extract arterial measurements from digitized tissue samples often focus on area-based metrics, such as quantifying specific components within an artery—namely, the lumen, intima, and media—and calculating relative ratios among areas of different tissue compartments.^6^^,^^28^^,^^29^ Although these techniques provide key insight into the automatic characterization of arteriosclerosis, they are often considered oversimplifications of the underlying biological complexities and may fail to provide generalized prognostic value.^6^ Furthermore, during tissue preparation, sectioning, and staining processes, the morphology of arteries may deform, which limits the accuracy of area-based measurements in capturing arterial wall thickening. In addition, the tissue sectioning process may cut arteries at various locations and angles, leading to irregular arterial appearances, such as open lumen at the edges of the tissue and multiple instances of arterial bifurcations at tangential cuts. These irregularities can also result from partial cuts and tissue folding or wrinkling, further complicating the assessment of arteriosclerosis.^30^

In this work, we address the challenge of arterial and arteriolar characterization by introducing a novel hand-crafted computer vision approach that uses two-dimensional (2D) ray casting and radial sampling to systematically measure intima and media thickness. Our method represents both media and intima thickness as a function of spatially encoded polar coordinates along the entire arterial perimeter. As a result, signal-smoothing techniques can effectively mitigate the impact of variable arterial morphology caused during tissue harvesting and preparation. Moreover, our approach is adept at analyzing arteries with irregular appearances, such as edge artifacts and arterial bifurcations, by identifying and discarding corrupted radial samples and using the remaining samples for a more robust representation. To demonstrate the feasibility of our technique, we first provide a numerical validation of our approach based on simulated arterial shape distortion. Subsequently, we applied our approach to WSIs from 33 patients’ kidney biopsies, developing a multi-class deep learning (DL) model for intra-arterial segmentation and evaluating our thickness measurement technique based on both manual and DL segmentation.

## 2 Method

### 2.1 Generalized Measurements of Intra-Arterial Thickness

#### 2.1.1 Thickness measurement based on radial sampling

The central challenge in measuring intima and media thickness arises from their non-uniform distribution and variability across different locations within an artery. To address this issue, we employed radial sampling and made radial measurements of media and intima thickness as a function of spatially encoded polar coordinates throughout the artery. In addition, arterial morphology can vary widely throughout the WSI based on sampling effects. These include (i) edge cuts, which result in an open lumen, and (ii) tangential cuts, which result in the presence of multiple, distinct areas of the lumen and intima. To address this issue, our approach selectively excludes specific radial measurements while retaining the remainder for analysis. Our technique for handling these three common arterial appearances is as follows.

##### Appearance 1: three nested layers of arterial sub-compartments from orthogonal tissue cuts

In an orthogonal cross-section, four arterial sub-compartments are visible: adventitia (excluded from this analysis because outside boundary cannot be separated from interstitial collagen histologically), media, intima, and lumen. Denoting the outer contours of media, intima, and lumen as $C_{m}$, $C_{i}$, and $C_{l}$, respectively, we first calculate the luminal center of mass (CoM) by leveraging the spatial moments of $C_{l}$, as shown in Eq. (1)

$$\mathrm{CoM}=(\frac{M_{10}}{M_{00}},\frac{M_{01}}{M_{00}}),$$ (1)

where the spatial moment $M_{ij}=\sum_{(x,y)\in C_{l}}(x^{i}y^{j})$.

**Fig. 1:**
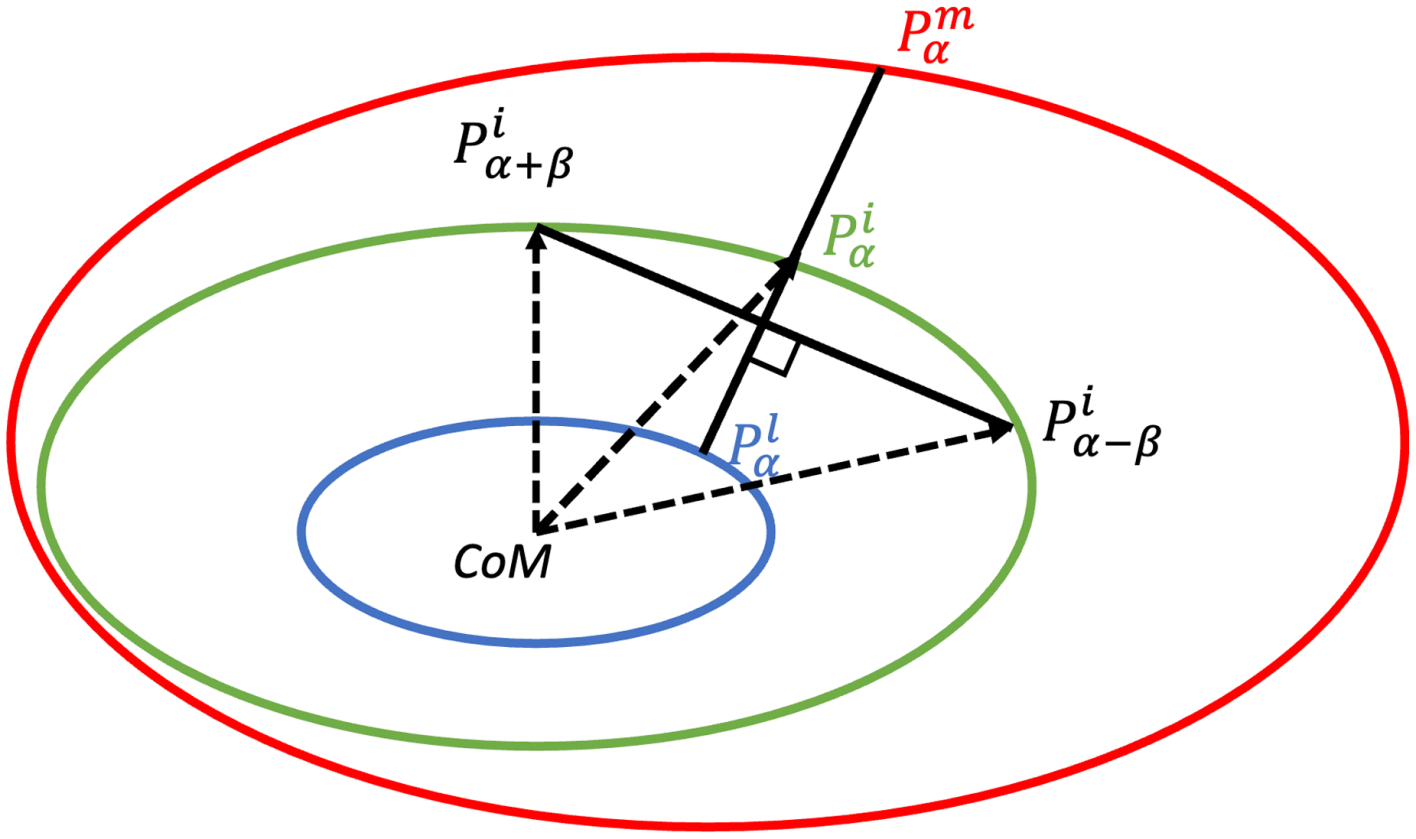
Illustration for the radial measurement at angle α. The media, intima, and lumen outer contours are depicted in red, green, and blue, respectively. Intersections between $C_{i}$ and rays $R_{\alpha -\beta }$, $R_{\alpha }$, and $R_{\alpha +\beta }$ are marked as $P_{\alpha -\beta }^{i}$, $P_{\alpha }^{i}$, and $P_{\alpha +\beta }^{i}$. $P_{\alpha }^{m}$ and $P_{\alpha }^{l}$ mark the intersections of the line perpendicular to the vector $\left(P_{\alpha -\beta }^{i},P_{\alpha +\beta }^{i}\right)$ with $C_{m}$ and $C_{l}$. Media and intima thickness at angle α, denoted as $T_{\mathrm{media}}^{\alpha }$ and $T_{\mathrm{intima}}^{\alpha }$, respectively, were calculated as the Euclidean distance of $P_{\alpha }^{m}-P_{\alpha }^{i}$ and $P_{\alpha }^{i}-P_{\alpha }^{l}$, respectively.

**Fig. 2:**
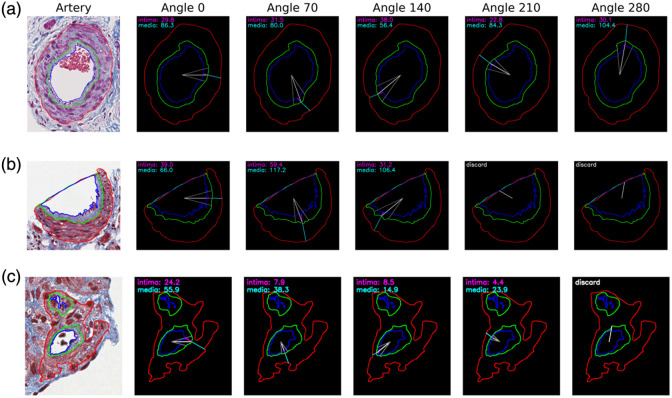
Illustrating examples of radial measurements at various polar coordinates. The first column shows images of the arteries cropped from trichrome-stained WSI, sourced from the NEPTUNE digital pathology repository, overlaid with geometric annotations. Columns 2 to 6 present auxiliary lines illustrating thickness measurements at angles 0, 70, 140, 210, and 280 deg. (a) Appearance 1: three nested layers of arterial sub-compartments from orthogonal issue cuts. Cross-sectional view of a complete artery. Thickness is measured at all five specified angles. (b) Appearance 2: open lumen from edge tissue cuts. Incomplete artery at the edge of the biopsy core. Measurements at angles 210 and 280 deg are discarded as they fall within the range of open lumen. (c) Appearance 3: multiple areas of lumen/intima from tangential tissue cuts. Serpiginous artery cuts through two adjacent points, resulting in a dual lumina connected by a media layer. The radial measurement at angle 280 deg was excluded due to its intersection with other lumen and intima regions.

As depicted in Fig. 1, for a given radial measurement at angle α, we cast three rays $R_{\alpha -\beta }$, $R_{\alpha }$, and $R_{\alpha +\beta }$, from CoM at angle α−β, α, and α+β, respectively, and calculate their intersections with $C_{i}$ as $P_{\alpha -\beta }^{i}$, $P_{\alpha }^{i}$, and $P_{\alpha +\beta }^{i}$. In cases of multiple intersections, we choose the farthest. From $P_{\alpha }^{i}$, the line perpendicular to the vector $\left(P_{\alpha -\beta }^{i},P_{\alpha +\beta }^{i}\right)$ was identified as $L_{\alpha }$, and its intersections with $C_{m}$ and $C_{l}$ were calculated as the points $P_{\alpha }^{m}$ and $P_{\alpha }^{l}$, respectively. In cases of multiple intersections, we choose the closest for $C_{m}$ and $C_{l}$. Media and intima thickness at angle α, denoted as $T_{\mathrm{media}}^{\alpha }$ and $T_{\mathrm{intima}}^{\alpha }$, respectively, were calculated as the Euclidean distance of $P_{\alpha }^{m}-P_{\alpha }^{i}$ and $P_{\alpha }^{i}-P_{\alpha }^{l}$, respectively. Figure 2(a) illustrates the radial measurements at various polar coordinates within the artery.

##### Appearance 2: open lumen from edge tissue cuts

When the kidney biopsy needle penetrates the kidney parenchyma, it may cut through the arteries, appearing incomplete at the edge of the kidney biopsy core and resulting in “partial open lumens.” For arteries with open lumens, we first identify the radial samples falling within the range of the open lumen, which are samples with intima and media thickness values below a pre-defined threshold. We then exclude samples both within and adjacent to these open lumen areas. This is achieved by employing a moving window technique, whereby we discard all samples within a certain window if the count of open lumen samples exceeds a specific limit. Figure 2(b) illustrates the radial measurements for an artery with the open lumen at the edge of the tissue, and radial measurements at angles 210 and 280 deg were discarded as they fall within the range of open lumen.

##### Appearance 3: multiple areas of lumen/intima from tangential tissue cuts

Multiple areas of lumen or intima can arise due to various factors, such as tissue folding, wrinkling during the cutting or mounting process, and bifurcation points in the artery where a single vessel splits into multiple smaller vessels. In cases where multiple lumens or intima are present, the lumen–intima–media combination with the largest intima and lumen area is selected for thickness measurements. Samples, where $R_{\alpha }$ intersects with other lumens or intima within the artery, and adjacent samples within a pre-defined window are discarded. Figure 2(c) illustrates the radial measurements for an artery with multiple areas of lumen/intima. In this instance, only the lower intima–lumen region was utilized for analysis, and the radial measurement at angle 280deg was discarded due to its intersection with other lumen and intima regions.

#### 2.1.2 Filtering, missing value imputation, and normalization

##### Filtering

To address the challenges posed by the distorted shapes and varying thicknesses in arterial measurements, we implemented a two-step filtering process. First, a moving median filter was applied to mitigate the impact of outliers, where each thickness measurement is replaced by the median value of its neighbors within a specific window size. Subsequently, a moving average filter was employed to further smooth the measurements and reduce noise, where each thickness measurement is replaced with the average value of its neighbors within the same window size.

##### Missing value imputation

During the radial measurement process, instances may arise where $L_{\alpha }$ does not intersect with $C_{l}$, leading to missing measurement values. Instead of removing these sample points, we applied a weighted imputation method to the radial measurements of media and intima thickness. Given the missing value $l_{i}$ at angle i in the list of measurements l, we first identified its closest non-missing values in the left and right, denoted as $l_{\mathrm{left}}$ and $l_{\mathrm{right}}$, respectively. Then, we impute the missing value by taking a weighted average of $l_{\mathrm{left}}$ and $l_{\mathrm{right}}$, as shown in Eq. (2)

$$l_{i}=\frac{w_{\mathrm{left}}*l_{\mathrm{left}}+w_{\mathrm{right}}*l_{\mathrm{right}}}{\left(w_{\mathrm{left}}+w_{\mathrm{right}}\right)},$$ (2)

where $w_{\mathrm{left}}=\frac{1}{\left|\mathrm{left}-i\right|}$ and $w_{\mathrm{right}}=\frac{1}{\left|\mathrm{right}-i\right|}$.

##### Normalization

The radial measurements of media and intima thickness are based on Euclidean distances among pixel coordinates, with values dependent on image resolution and artery size. To account for these factors, we normalize the media and intima thickness values by dividing them by the artery wall thickness median value.

**Fig. 3:**
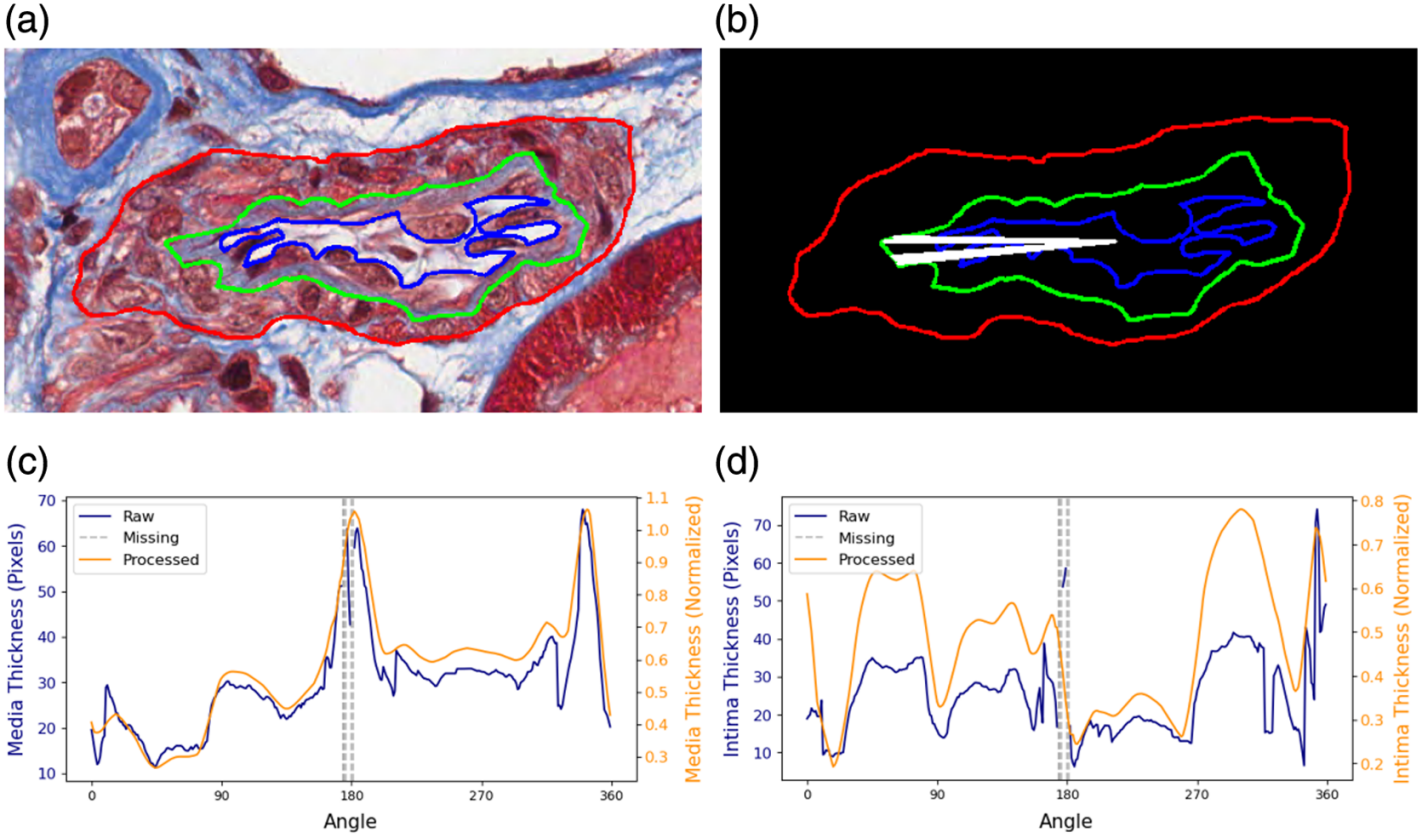
Illustration of the application of filtering, missing value imputation, and normalization. (a) Artery: an artery with overlaid sub-component annotations. (b) Annotations: annotations of the outer contours of the media, intima, and lumen in distinguishable colors of red, green, and blue, respectively. Measurements at angle α∈[174°,176°]∪[180°,181°] are missing, marked by white radial lines. (c) Media thickness: radial measurements of media thickness, presented as a function of angle, before and after application of the processing steps. (d) Intima thickness: radial measurements of intima thickness, before and after the processing stages.

Figure 3 provides an example of the radial measurements before and after steps of filtering, missing value imputation, and normalization for an artery with missing values at angle α∈[174  deg,176  deg]∪[180  deg,181  deg].

#### 2.1.3 Feature extraction

Based on the radial measurements of intima and media thickness, we further computed the intima-to-media ratio at angle α, denoted as $R_{\mathrm{intima}-\mathrm{media}}^{\alpha }$, defined as Eq. (3)

$$R_{\mathrm{intima}-\mathrm{media}}^{\alpha }=\frac{T_{\mathrm{intima}}^{\alpha }}{T_{\mathrm{intima}}^{\alpha }+T_{\mathrm{media}}^{\alpha }}.$$ (3)

As a result, our data are represented by three distinct series of measurements, each corresponding to a specific attribute: intima thickness, media thickness, and the intima-to-media ratio. These measurements are taken in a circular manner along the arterial contour, where the first and last samples are adjacent. To further characterize the morphological appearance of an artery, we extract two categories of pathomic features from each list: (i) global features, which describe the overall distribution of intra-arterial thickness, and (ii) local features, which describe the local variations of distribution of arterial thickness.

##### Global features

We calculate the global statistical features, including the average, median, and variance for each set of measurements.

##### Local features

**Fig. 4:**
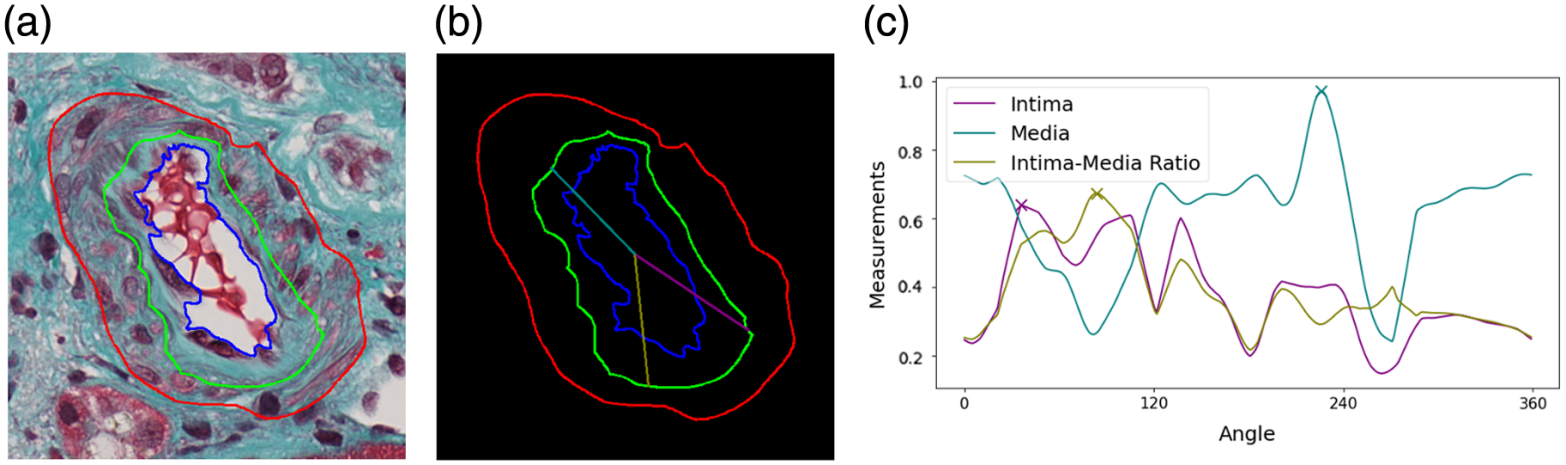
Illustration of local feature extraction. (a) Artery: an artery with overlaid sub-component annotations. (b) Annotations: annotations of the outer contours of the media, intima, and lumen in distinguishable colors of red, green, and blue, respectively. Radial lines highlight angles 43, 227, and 89 deg, corresponding to local peaks in measurements of intima thickness, media thickness, and intima–media ratio, respectively. (c) Thickness measurements: radial measurements for intima thickness, media thickness, and intima–media ratio, with the peaks denoted by X markers at angles 36, 226, and 84 deg, respectively.

We extracted local features by analyzing the peak properties within each series of measurements. Peaks are identified as local maxima satisfying the width greater than a pre-determined threshold $\theta_{w}$. The maximum height and prominence of the peak properties are utilized as local features for analysis. Figure 4 provides an example of the detected peaks at angles 43, 227, and 89 deg for intima thickness, media thickness, and the intima-to-media ratio, respectively.

#### 2.1.4 Numerical validation

As discussed in Sec. 1, the processes of tissue harvesting and preparation can cause arterial shape distortion. To study the effects of these distortions on our measurement technique, we conducted a numerical analysis by simulating arteries with three different appearances and by representing them with different geometric shapes, such as circles and semi-circles. We introduced random distortions using elastic deformation to systematically simulate variations in arterial morphology, from which to evaluate our measurement technique.

**Fig. 5:**
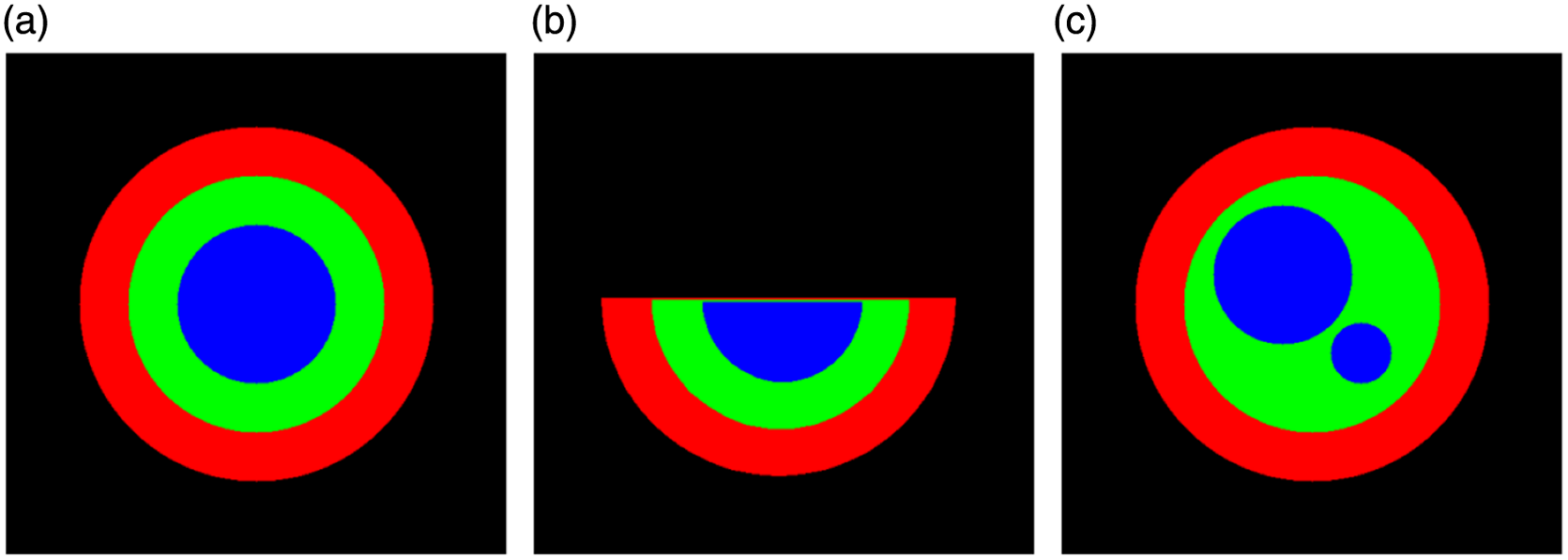
Illustrations of simulated artery appearances. (a) Three nested layers: cross-sectional view of a complete artery. (b) Open lumen: artery with an open lumen appearance. (c) Multiple areas of lumen: artery with the appearance of multiple areas of lumen.

Specifically, for an artery exhibiting a regular appearance, we simulated the initial artery using concentric circles $c_{m},c_{i},c_{l}$ with different radii $r_{m},r_{i},r_{l}$ representing the outer contours of the media, intima, and lumen, respectively, where $r_{m}>r_{i}>r_{l}$. In the case of an artery with an open lumen, we used three semi-circles to model the contours. For an artery with multiple intima/lumens, we employed four circles, with two distinct circles representing the lumens. Representative illustrations of these simulated artery appearances can be found in Fig. 5.

Subsequently, we applied random distortions to these initial geometries using elastic deformation. We generated smooth deformations by creating random displacement fields at the horizontal and vertical directions for each pixel in the image. To study the effects of different levels of distortion, we scaled these displacement fields by a factor λ, where λ controls the level of distortion, with larger values of λ resulting in more substantial distortions. We varied the distortion order λ from 0 to 3000 in increments of 500. The displacement fields were subsequently smoothed using a Gaussian function with a standard deviation of 30 pixels. Per-pixel displacements were then computed using linear interpolation. For each deformation-specific iteration, we applied our measurement technique and analyzed trends as a function of deformation magnitude.

### 2.2 Integrating Multi-Class Deep Learning Segmentation and Intra-Arterial Thickness to Quantify Arteriosclerosis

To demonstrate the effectiveness of the proposed thickness measurement approach, we applied the proposed thickness measurement technique to patient data, based on both manual and DL-based segmentation of intra-arterial compartments. The study received ethical approvals from the boards of the NEPhrotic syndrome sTUdy NEtwork (NEPTUNE) and Cure Glomerulonephropathy (CureGN) consortia, as well as from the Institutional Review Board of the University of Michigan.

#### 2.2.1 Whole slide imaging dataset

Our work leverages 753 arteries identified from 33 trichrome-stained WSIs, scanned at a magnification of ×40, which is a ∼0.25-μm/pixel isotropic resolution. Each WSI is from a distinct patient, including 20 from the NEPTUNE,^31^ 9 from the CureGN,^32^ and 4 from the University of Michigan digital pathology repositories.

#### 2.2.2 Arteriosclerosis scoring and manual segmentation

The severity of the intima fibrosis and narrowing of the lumina as a measure of arteriosclerosis was scored semiquantitatively (0 to 3) at the individual artery level by two renal pathologists (DD and JBH) using a standard clinical definition (Table 1). In addition, the media, intima, and lumen were manually segmented for each artery using QuPath.^33^

**Table 1:** Arteriosclerosis scores based on vascular narrowing of the luminal area.

Score	Luminal area narrowing
0: without arteriosclerosis	None
1: mild arteriosclerosis	Up to 25%
2: moderate arteriosclerosis	25% to 50%
3: severe arteriosclerosis	More than 50%

#### 2.2.3 Multi-class deep learning segmentation of intra-arterial compartments

The proposed approach for measuring intra-arterial thickness relies on the accurate segmentation of the media, intima, and lumen. Manual segmentation of these components, however, can be meticulous and time-consuming. Therefore, to illustrate the versatility and effectiveness of our measurement method, we have developed a multi-class DL framework for automating this segmentation process. This inclusion of DL segmentation serves to demonstrate that our measurement approach is robust and can be effectively applied in both manually segmented and DL-augmented workflows, ensuring its broad applicability.

##### Architecture design

We employed a nine-module convolutional neural network based on the common U-Net architecture with a dilated bottleneck, which has been proved effective in the segmentation of histologic structures on kidney biopsies.^26^^,^^34^ The network’s input accepts 256×256×3 red, green, blue (RGB) images, outputting a 256×256×4 segmentation map for the background, media, intima, and lumen.

##### Model training

We used 648 artery images, derived from 20 WSIs from NEPTUNE and four WSIs from U MICHIGAN to train the DL model. To boost model generalization, we applied a data augmentation procedure (e.g., horizontal vertical flip and crop resize). In addition, we incorporated transfer learning by initializing the first four fully convolutional blocks of the network with pre-trained weights from the publicly available VGG16 model, originally trained on the ImageNet dataset. During the training process, a cross-entropy loss function was minimized based on Adam optimization to learn optimal model parameters. The training was run for 50 epochs with a batch size of 4 and an initial learning rate of 0.001.

##### Post-processing

After acquiring the DL segmentation output and applying an argmax operation, we obtain three distinct Boolean masks representing the segmented artery sub-compartments: media, intima, and lumen. To enhance the quality of these segmentation results, we implement a noise reduction step on each mask by removing small holes and objects with areas below 5% of the total positive pixels. From the cleaned segmentation masks, we identify the largest contour in each mask as the input for our proposed thickness measurement technique.

#### 2.2.4 Evaluation and performance metrics

We utilized 105 artery images (nine WSIs from CureGN) to evaluate the ability of the extracted features from our thickness measurements to quantify arteriosclerosis. The evaluation was based on the segmentation by both a renal pathologist and the developed deep learning model.

##### Individual feature correlation analysis

We first measure the association between each extracted feature and arteriosclerosis severities by calculating Kendall’s tau-b correlation coefficient, τ. τ is a statistic used to measure the ordinal association between two measured quantities. Despite our features being continuous, they can be conceptualized as ordinal for this analysis due to their ranking nature. Note that τ takes values between −1 and +1. A value of ±1 indicates a perfect association between the feature and arteriosclerosis severities, whereas a coefficient closer to 0 suggests a weaker relationship.

##### Multi-variate feature evaluation

To evaluate the ability to quantify arteriosclerosis of our thickness-based features in comparison with the baseline area-based features, we employed the ridge regression estimator. We compute ridge regression estimators using three distinct feature sets: only area-based features, only thickness-based features, and a combination of both. The performance and contribution of these features were then assessed using the $R^{2}$ score of the corresponding estimator. $R^{2}$ score ranges between 0 and 1, denoting the effectiveness of the chosen features for quantifying arteriosclerosis.

## 3 Experiments and Result

### 3.1 Numerical Validation

**Fig. 6:**
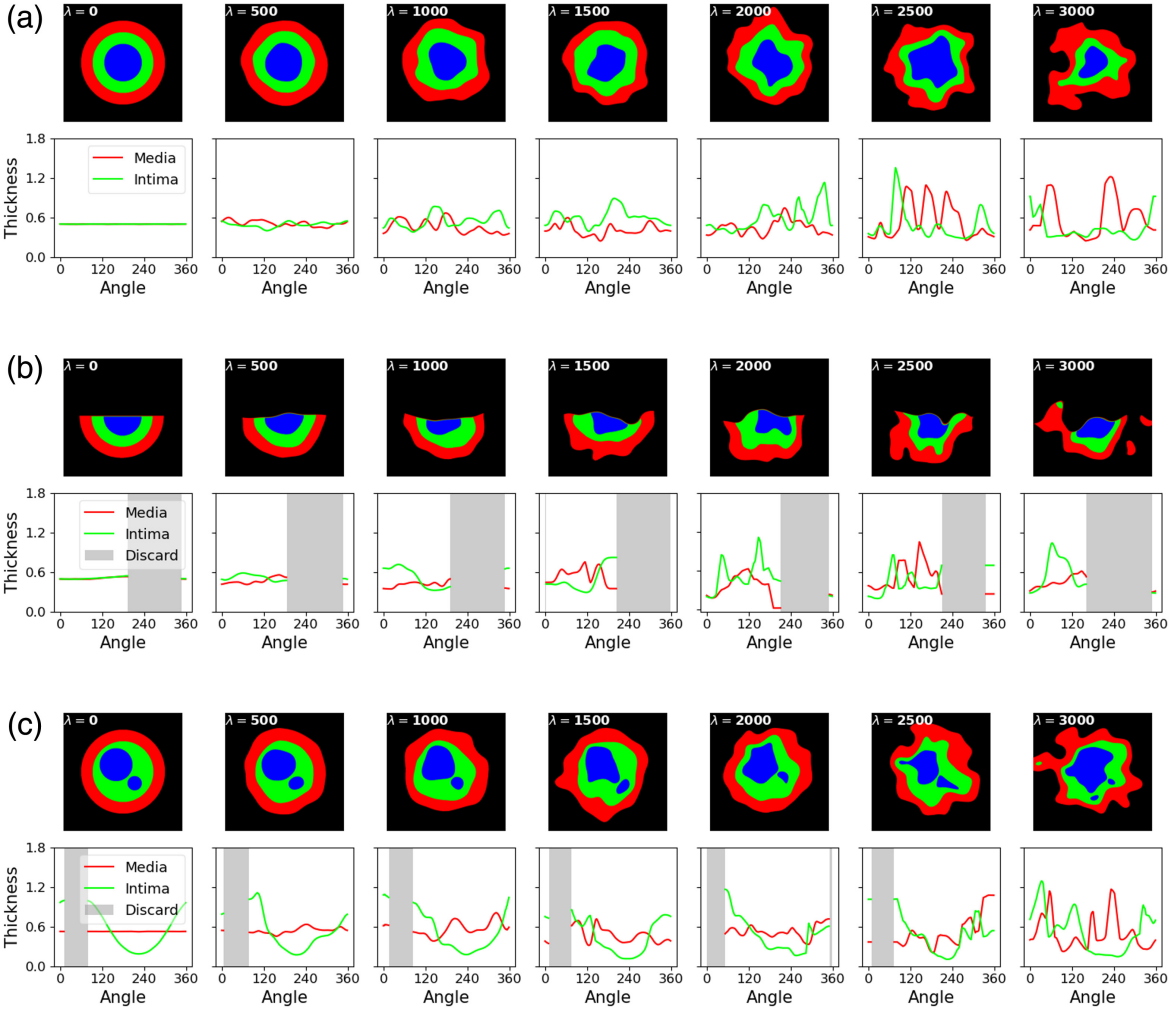
Numerical validation for arteries of different appearances. For each subfigure, the top row shows the same artery under varying shape distortions, whereas the bottom row presents the radial measurements of intima and media thickness, plotted against the angle, for each distorted artery. (a) Appearance 1: three nested layers of arterial sub-compartments. Artery simulation for the appearance of three nested layers of arterial sub-compartments. As distortion levels rise, our measurement technique effectively captures the decreasing smoothness in the intima and media thickness. (b) Appearance 2: open lumen. Artery simulation for the open lumen appearance. Apart from capturing smoothness variations, our approach can identify and discard measurements that fall within the range of an open lumen. (c) Appearance 3: multiple areas of lumen/intima. Artery simulation for the appearance of multiple areas of lumen/intima. Measurements that intersect other lumen areas are excluded.

Figure 6 shows the simulated arteries for different appearances under various deformation orders, along with the results of intima–media thickness measurements. For all three appearances, the simulated arteries present increasing deformation as the distortion order λ increases, leading to a less smooth representation of measured intima and media thickness with respect to angle. In the case of an open lumen appearance, our approach can identify and discard measurements that fall within the range of the open lumen. For the simulations on the appearance of multiple areas of lumen/intima, our approach can recognize and exclude measurements that intersect other lumen areas.

### 3.2 Intra-Arterial Thickness-Based Features to Quantify Arteriosclerosis

#### 3.2.1 Individual feature correlation analysis

##### Manual segmentation

**Fig. 7:**
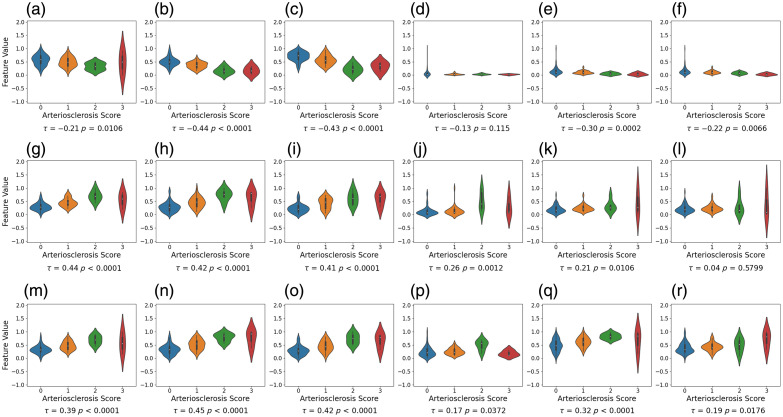
Violin plots representing the distribution of feature values for each arteriosclerosis severity score with associated Kendall’s coefficients, where features are derived from thickness measurement based on manual segmentation and normalized using min–max scaling. The top, middle, and bottom rows represent the features extracted from media thickness, intima thickness, and intima–media thickness ratio, respectively. The intima area feature exhibits a clear separability with τ=0.44. Global thickness-based features (average and median derived from all three measurements) present a comparative separability (|τ|≥0.4). Local features (peak height from media and ratio measurements) present a moderate association, with |τ|≥0.30. (a) Media area (τ=−0.21, p=0.0106). (b) Media average (τ=−0.44, p<0.0001). (c) Media median (τ=−0.43, p<0.0001). (d) Media variance (τ=−0.13, p=0.115). (e) Media peak height (τ=−0.30, p=0.0002). (f) Media peak prominence (τ=−0.22, p=0.0066). (g) Intima area (τ=0.44, p<0.0001). (h) Intima average (τ=0.42, p<0.0001). (i) Intima median (τ=0.41, p<0.0001). (j) Intima variance (τ=0.26, p=0.0012). (k) Intima peak height (τ=0.21, p=0.0106). (l) Intima peak prominence (τ=0.04, p=0.5799). (m) Ratio intima/media area (τ=0.39, p<0.0001). (n) Ratio average (τ=0.45, p<0.0001). (o) Ratio median (τ=0.42, p<0.0001). (*p*) Ratio variance (τ=0.17, p=0.0372). (q) Ratio peak height (τ=0.32, p<0.0001). (r) Ratio peak prominence (τ=0.19, p=0.0176).

We evaluated the feature values derived from thickness measurements using manual segmentation (Fig. 7). For the area-based features, the intima area feature exhibits a clear separability with τ=0.44. For the proposed thickness-based features, the global features (average and median) derived from all three measurements present a comparative separability compared with the intima area feature, with |τ|≥0.4. Among the local features, the peak height from media and ratio measurements show a moderate association, with |τ|≥0.3.

##### DL segmentation

**Fig. 8:**
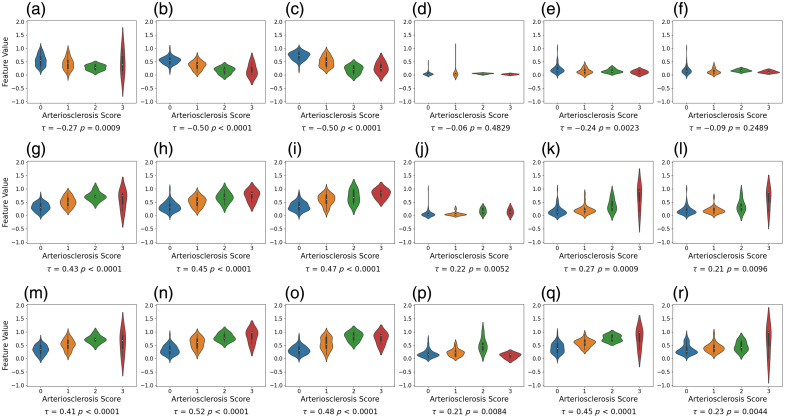
Violin plots representing the distribution of feature values for each arteriosclerosis severity score with associated Kendall’s coefficients, where features are derived from thickness measurement based on deep learning segmentation and normalized using min–max scaling. The top, middle, and bottom rows represent the features extracted from media thickness, intima thickness, and intima–media thickness ratio, respectively. Six global features (average and median from all three measurements) and one local feature (ratio peak height) present a more robust association compared with the intima area feature. (a) Media area (τ=−0.27, p=0.0009). (b) Media average (τ=−0.50, p<0.0001). (c) Media median (τ=−0.50, p<0.0001). (d) Media variance (τ=−0.06, p=0.4829). (e) Media peak height (τ=−0.24, p=0.0023). (f) Media peak prominence (τ=−0.09, p=0.2489). (g) Intima area (τ=0.43, p<0.0001). (h) Intima average (τ=0.45, p<0.0001). (i) Intima median (τ=0.47, p<0.0001). (j) Intima variance (τ=0.22, p=0.0052). (k) Intima peak height (τ=0.27, p=0.0009). (l) Intima peak prominence (τ=0.21, p=0.0096). (m) Ratio intima/media area (τ=0.41, p<0.0001). (n) Ratio average (τ=0.52, p<0.0001). (o) Ratio median (τ=0.48, p<0.0001). (p) Ratio variance (τ=0.21, p=0.0084). (q) Ratio peak height (τ=0.45, p<0.0001). (r) Ratio peak prominence (τ=0.23, p=0.0044).

Our DL segmentation model effectively delineates the intra-arterial compartments, achieving Dice scores of 0.97 for the background, 0.84 for the media, 0.78 for the intima, and 0.86 for the lumen. Subsequently, we evaluated the feature values derived from thickness measurements based on the segmentation model (Fig. 8). For baseline area-based features, the intima area exhibits a clear separability with τ=0.43. For the proposed thickness-based features, six global features (average and median from all three measurements) and one local feature (ratio peak height) present a more robust association compared with the intima area feature. It is notable that the ratio average displayed a strong separability, with τ=0.52.

**Fig. 9:**
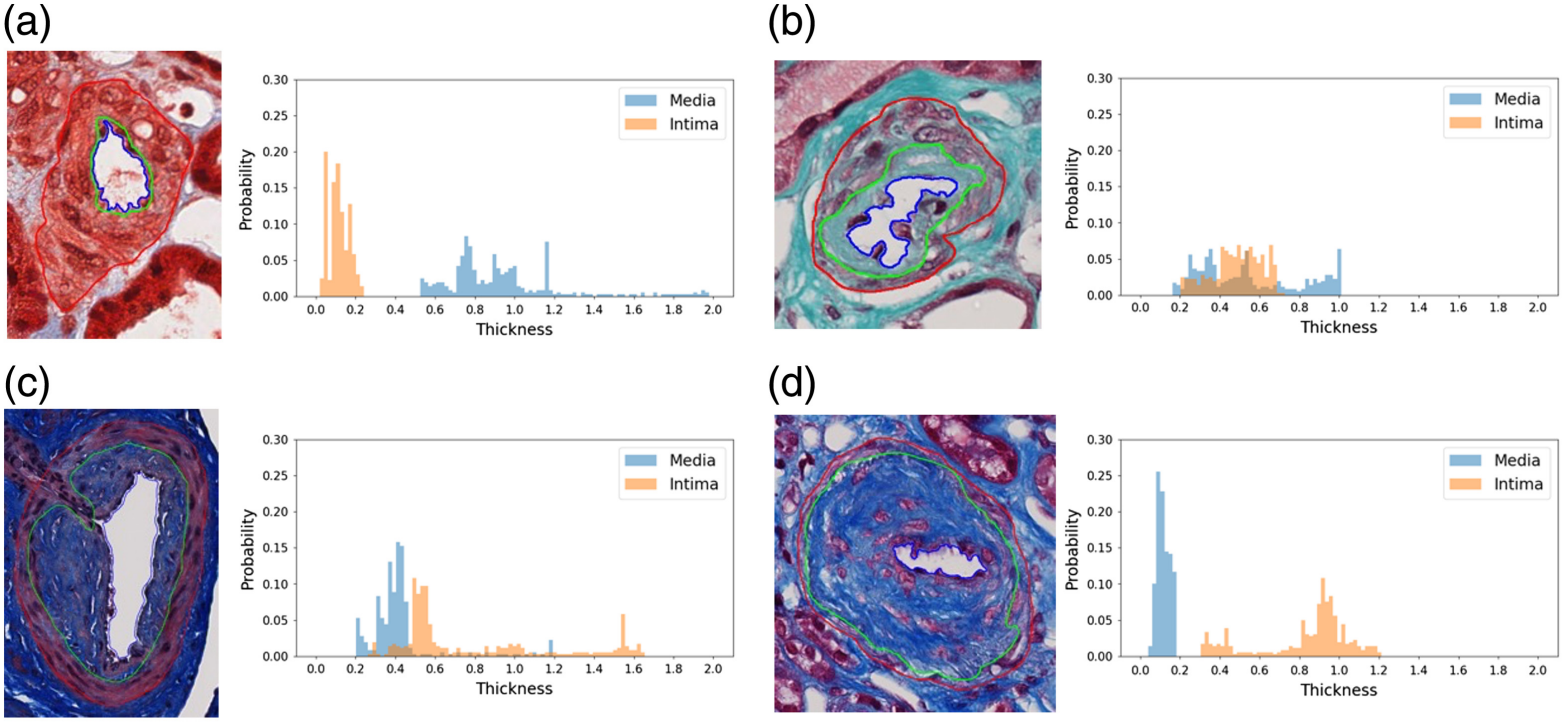
Distribution of intima–media thickness measurements across arteriosclerosis severity scores. (a) Artery example with score 0: intima and media thickness distributions are distinct, with media values substantially larger. (b) Artery example with score 1: intima and media thickness distributions overlap, resulting in similar thickness values for both. (c) Artery example with score 2: intima thickness values marginally surpass those of the media. (d) Artery example with score 3: intima and media thickness distributions separated again, but with intima values significantly exceeding media values.

These results suggest that the proposed thickness-based features especially global features can individually effectively contribute to the characterization of arteriosclerosis severity. When the score is 0, the distributions of intima and media thickness are well-separated, with the media exhibiting considerably larger values, indicating a normal artery. As the score increases, there is a noticeable shift in the intima thickness distribution toward the right, signaling the progression of arteriosclerosis severities. Examples of the intima–media thickness distributions for arteries with arteriosclerosis scores ranging from 0 to 3 are illustrated in Fig. 9.

#### 3.2.2 Multi-variate feature evaluation

The $R^{2}$ scores derived from ridge regression estimators using different feature sets are shown in Table 2. First, we observed that the $R^{2}$ score for quantifying arteriosclerosis exhibits minimal difference between features derived from manual segmentation and those from DL segmentation, indicating that both methods are comparably effective when used as segmentation techniques for feature extraction. Secondly, incorporating thickness-based features greatly improves the performance of quantifying arteriosclerosis, with the $R^{2}$ score rising from 0.3 to 0.5, which emphasizes the potential of the proposed thickness-based features in quantifying arteriosclerosis.

**Table 2:** Comparison of $R^{2}$ scores using the ridge regression estimator across various feature sets and segmentation methods for quantifying arteriosclerosis.

Feature sets	Manual segmentation	DL segmentation
Area-based features only	0.314	0.311
Thickness-based features only	0.498	0.509
Combination of both	0.514	0.511

### 3.3 Ablation Studies

#### 3.3.1 Impact of filtering

**Fig. 10:**
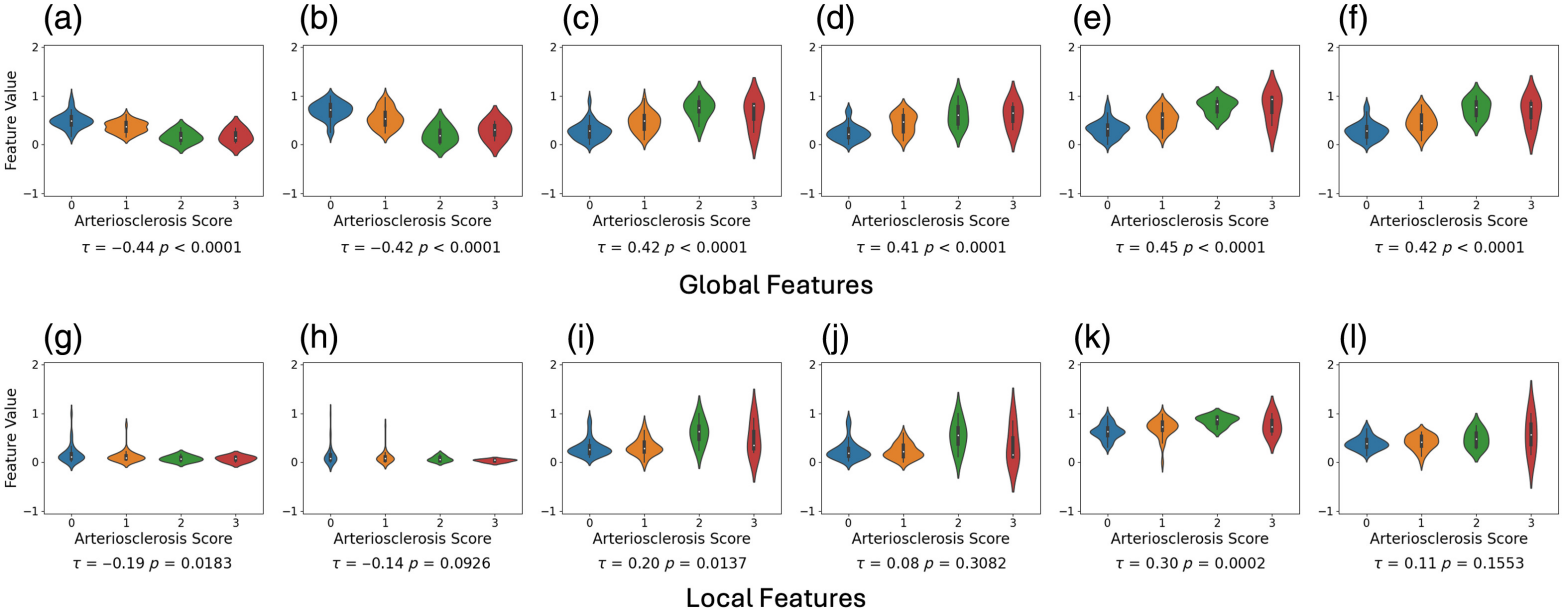
Violin plots representing the distribution of feature values for each arteriosclerosis severity score with associated Kendall’s coefficients, where features are derived from thickness measurement based on manual segmentation and normalized using min–max scaling, without filtering in the post-processing. The analysis shows that without filtering, five out of six local features exhibit a decline in performance compared with filtered signal. Notably, the separability provided by the media peak height metric drops from |τ|=0.30 to |τ|=0.19. (a) Media average (τ=−0.44, p<0.0001). (b) Media median (τ=−0.42, p<0.0001). (c) Intima average (τ=0.42, p<0.0001). (d) Intima median (τ=0.41, p<0.0001). (e) Ratio average (τ=0.45, p<0.0001). (f) Ratio median (τ=0.42, p<0.0001). (g) Media peak height (τ=−0.19, p=0.0183). (h) Media peak prominence (τ=−0.14, p=0.0926). (i) Intima peak height (τ=0.20, p=0.0137). (j) Intima peak prominence (τ=0.08, p=0.3082). (k) Ratio peak height (τ=0.30, p=0.0002). (l) Ratio peak prominence (τ=0.11, p=0.1553).

This subsection evaluates the impact of filtering on feature performance. Without filtering, while global features maintain similar performance levels, five out of six local features exhibit a decline in performance (Fig. 10). Notably, the separability provided by the media peak height metric drops from |τ|=0.30 to |τ|=0.19.

#### 3.3.2 Impact of missing value imputation

**Fig. 11:**
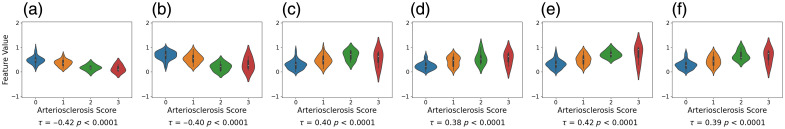
Violin plots representing the distribution of global feature values for each arteriosclerosis severity score with associated Kendall’s coefficients, where features are derived from thickness measurement based on manual segmentation and normalized using min–max scaling, without missing value imputation in the post-processing. The analysis shows that all six local features exhibit a decline in performance without imputation. Notably, the separability provided by intima–media ratio average metric decreases from τ=0.45 to τ=0.42. (a) Media average (τ=−0.42, p<0.0001). (b) Media median (τ=−0.40, p<0.0001). (c) Intima average (τ=0.40, p<0.0001). (d) Intima median (τ=0.38, p<0.0001). (e) Ratio average (τ=0.42, p<0.0001). (f) Ratio median (τ=0.39, p<0.0001).

In our test dataset, each artery has an average of 22 missing values out of a potential 360. This accounts for approximately 6.1% of the data points per artery being unusable due to missing values. Without imputation, the signals are discontinuous, and local features cannot be calculated. This subsection evaluates the performance of global features without missing value imputation. The results indicate a significant drop in performance across all six features when imputation is not applied (Fig. 11).

### 3.4 Inter-Observer Variability

**Fig. 12:**
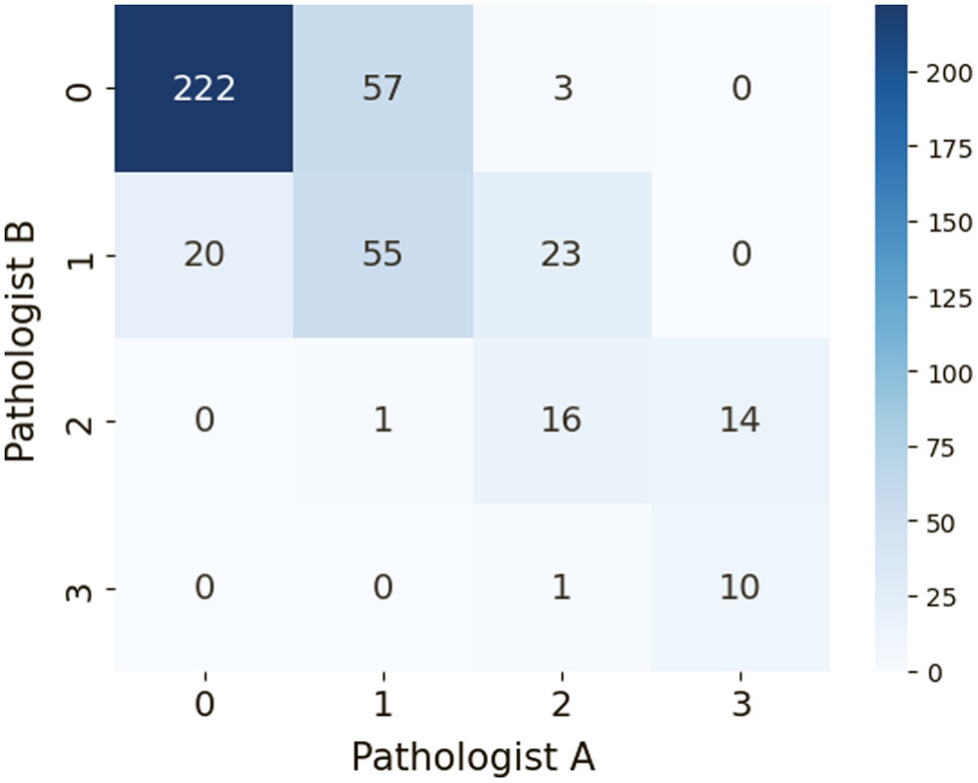
Confusion matrix comparing the arteriosclerosis scoring between two pathologists.

To evaluate the inter-observer variability of semi-quantitative, visual assessment of arteriosclerosis by trained renal pathologists, we prepared a subset of 422 arteries from 10 trichrome-stained WSIs, sourced from the NEPTUNE digital pathology repositories.^31^ Each artery was independently scored on a scale of 0 to 3 by two renal pathologists (DD and JBH). To quantify the inter-observer agreement, we employed Cohen’s kappa statistic, which yielded a value of k=0.48, indicating moderate agreement between the two readers. The confusion matrix illustrating the scoring differences among the pathologists is presented in Fig. 12.

In addition, we calculated the $R^{2}$ value between the two sets of annotations, which was 0.45. This value is significantly lower compared with the results obtained from our proposed computational thickness measurement method (Table 2).

## 4 Discussion

The emergence of digital pathology and computational techniques has transformed the pathology landscape.^18^ Although methodologies for visual assessment have improved using WSIs over conventional bright-field microscopes, the visual assessment of the extent and severity of structural lesions relies on semiquantitative metrics and remains relatively subjective. Computational image analysis methodologies offer the opportunity to better quantify structural changes on WSIs.

In general, there are two primary options for interpreting digital pathology images: DL algorithms and hand-crafted feature extraction. In the medical field, DL has notably demonstrated its ability in tasks such as image segmentation, but its application in direct clinical diagnosis often presents challenges. DL features are typically latent variables generated from the transformation and encoding of the input data through the layers of the neural network. Although DL algorithms can uncover structural patterns without human guidance, they often have limitations such as the need for high-quality ground truth annotations, and extracted features lack explicit mathematical definitions.^34^^–^^37^ In contrast, hand-crafted feature extraction involves capturing and analyzing various features such as morphology, intensity, texture, higher-order, and spatial features.^18^ Hand-crafted features are mathematically well-defined and typically require domain knowledge for design and interpretation.

For this study, we chose to focus on developing a novel hand-crafted thickness measurement technique due to the inherent advantages it offers in terms of interpretability and applicability. Thus, we proposed a hand-crafted computer vision approach that systematically quantifies changes in the intima and media thickness of arteries and arterioles from digital kidney biopsies using 2D ray casting and radial sampling. Our approach contains elements of innovation and offers several advantages. First, it presents the thickness of both the media and intima as a function of spatially encoded polar coordinates along the entire arterial perimeter. Second, it is adept at analyzing arteries with irregular appearances, such as edge artifacts and arterial bifurcations, by identifying and discarding corrupted radial samples and using the remaining samples for a more robust analysis. Consequently, using these signal-smoothing techniques, we can effectively address the issues of arterial deformation introduced during tissue harvesting and preparation. Furthermore, by detecting and discarding corrupted radial samples, our approach is capable of analyzing arteries with irregular appearances, such as open lumens at the edge of tissue and multiple instances of local intima/lumen from tangential cuts and arterial bifurcations.

To demonstrate the feasibility of our technique, we initially conducted a numerical validation based on simulated arterial shape distortion. As the deformation increases in the simulated arteries, our measurement technique adeptly captured the decreasing smoothness in the intima and media thickness. Subsequently, we applied our approach to patient data and demonstrated its effectiveness in two different scenarios, intra-arterial segmentation performed by a renal pathologist, and segmentation via a deep learning model. Our individual feature correlation analysis indicates that global features (such as average and median from all three measurements) and local features (such as ratio peak height) provide a clear separability among arteriosclerosis severity. Further collective feature evaluation demonstrates that incorporating our proposed set of thickness-based features can greatly improve the performance in quantifying arteriosclerosis.

Training a DL-based classification model, or a machine learning model augmented with feature selection from a broad array of features, offers a path forward. However, such approaches often sacrifice interpretability and/or a complete mechanistic understating of the underlying biology. In this study, we focused on quantifying arterial thickness via a highly interpretable metric and demonstrated clinical relevance via associations with arteriosclerosis visual lexicons used in clinical pathology. Future emphasis on data assimilation, where physics-informed models are integrated with purely data-driven solutions,^38^^,^^39^ may provide a more rigorous description of the biology, ultimately leading to better interpretation of DL^40^^,^^41^ and more informed computational phenotyping.

A limitation of this study is that the dataset size is limited and the association with clinical outcomes was not explored. Ongoing studies are focusing on validating our findings in larger cohorts, investigating the utility of additional features, and exploring the development of automated systems that integrate these features for clinical decision-making. Furthermore, future work will also focus on evaluating the association of these pathomic features with clinical outcomes and the prognostic value of our approach compared with standard-of-care visual assessment.

## 5 Conclusion

In this work, we introduced a novel computer vision approach to computationally characterize intra-arterial morphology using WSIs from digital pathology archives. This rigorous approach for the quantification of arteriosclerosis has been proven robust and should serve as a blueprint to measure vascular damage across organs and different datasets. Its potential generalizability is promising for future use in clinical research, trials, and practice.

## Supplementary Material



## Data Availability

The authors do not have the permission to share the dataset utilized in this study. The source code is available at github.com/code-by-jin/Measurements-of-Intra-Arterial-Thickness

## References

[1] Hassan N. A. et al., “Arterial stiffness, physical activity, and 25-hydroxyvitamin D in patients with kidney transplant,” J. Amer. Soc. Nephrol. 29(3), 927–938 (2018). JASNEU 1046-6673 10.1590/1806-9282.63.10.910

[r2] Issa N. et al., “Larger nephron size, low nephron number, and nephrosclerosis on biopsy as predictors of kidney function after donating a kidney,” Amer. J. Transpl. 19(7), 1989–1998 (2019). 10.1111/ajt.15259 PMC6591036 30629312

[3] Denic A. et al., “Larger nephron size and nephrosclerosis predict progressive CKD and mortality after radical nephrectomy for tumor and independent of kidney function,” J. Amer. Soc. Nephrol. 31(11), 2642–2652 (2020). JASNEU 1046-6673 10.1681/ASN.2020040449 32938650 PMC7608955

[4] Kobiyama K. Ley K., “Atherosclerosis: a chronic inflammatory disease with an autoimmune component,” Circul. Res. 123(10), 1118–1120 (2018). 10.1161/CIRCRESAHA.118.313816 PMC6298754 30359201

[5] Sethi S. et al., “A proposal for standardized grading of chronic changes in native kidney biopsy specimens,” Kidney Int. 91(4), 787–789 (2017). 10.1016/j.kint.2017.01.002 28314581

[r6] Denic A. et al., “Prognostic implications of a morphometric evaluation for chronic changes on all diagnostic native kidney biopsies,” J. Amer. Soc. Nephrol. 33(10), 1927–1941 (2020). JASNEU 1046-6673 10.1681/ASN.2022030234 PMC9528338 35922132

[7] Roufosse C. et al., “A 2018 reference guide to the Banff classification of renal allograft pathology,” Transplantation 102, 1795–1814 (2018). TRPLAU 0041-1337 10.1097/TP.0000000000002366 30028786 PMC7597974

[8] Aeffner F. et al., “The gold standard paradox in digital image analysis: manual versus automated scoring as ground truth,” Arch. Pathol. Lab Med. 141, 1267–1275 (2017). 10.5858/arpa.2016-0386-RA 28557614

[9] Adam B. Randhawa P. Chan S., “Banff initiative for quality assurance in transplantation (BIFQUIT): reproducibility of polyomavirus immunohistochemistry in kidney allografts,” Am. J. Transplant. 14, 2137–2147 (2014). 10.1111/ajt.12794 25091177 PMC4194133

[r10] Haas M. Sis B. Racusen L., “Banff 2013 meeting report: inclusion of C4d-negative antibody-mediated rejection and antibody-associated arterial lesions,” Am. J. Transplant. 14, 272–283 (2014). 10.1111/ajt.12590 24472190

[r11] Roberts C. Beitsch P. Litz C., “Interpretive disparity among pathologists in breast sentinel lymph node evaluation,” Am. J. Surg. 186, 324–329 (2003). AJOOA7 0096-6347 10.1016/S0002-9610(03)00268-X 14553843

[12] Roberts J. Jin F. Thurloe J., “High reproducibility of histological diagnosis of human papillomavirus-related intraepithelial lesions of the anal canal,” Pathology 47, 308–313 (2015). PTLGAX 1465-3931 10.1097/PAT.0000000000000246 25938361

[13] Pantanowitz L. et al., “Twenty years of digital pathology: an overview of the road travelled, what is on the horizon, and the emergence of vendor-neutral archives,” J. Pathol. Inf. 9, 40 (2018). 10.4103/jpi.jpi_69_18 PMC6289005 30607307

[r14] Retamero J. A. Aneiros-Fernandez J. Del Moral R. G., “Complete digital pathology for routine histopathology diagnosis in a multicenter hospital network,” Arch. Pathol. Lab. Med. 144(2), 221–228 (2020). APLMAS 0003-9985 10.5858/arpa.2018-0541-OA 31295015

[r15] Pantanowitz L. et al., “Validating whole slide imaging for diagnostic purposes in pathology: guideline from the College of American Pathologists Pathology and Laboratory Quality Center,” Arch. Pathol. Lab. Med. 137(12), 1710–1722 (2013). APLMAS 0003-9985 10.5858/arpa.2013-0093-CP 23634907 PMC7240346

[r16] Brachtel E. Yagi Y., “Digital imaging in pathology—current applications and challenges,” J. Biophotonics 5(4), 327–335 (2012). 10.1002/jbio.201100103 22213680

[r17] Baidoshvili A. et al., “Evaluating the benefits of digital pathology implementation: time savings in laboratory logistics,” Histopathology 73(5), 784–794 (2018). HISTDD 1365-2559 10.1111/his.13691 29924891

[r18] Barisoni L. et al., “Digital pathology and computational image analysis in nephropathology,” Nat. Rev. Nephrol. 16(11), 669–685 (2020). 10.1038/s41581-020-0321-6 32848206 PMC7447970

[r19] Tellez D. et al., “Quantifying the effects of data augmentation and stain color normalization in convolutional neural networks for computational pathology,” Med. Image Anal. 58, 101544 (2019). 10.1016/j.media.2019.101544 31466046

[20] Barisoni L. Hodgin J. B., “Digital pathology in nephrology clinical trials, research, and pathology practice,” Curr. Opin. Nephrol. Hypertens. 26(6), 450–459 (2017). CNHYEM 10.1097/MNH.0000000000000360 28858910 PMC5955389

[21] Wuttisarnwattana P. et al., “Automatic stem cell detection in microscopic whole mouse cryo-imaging,” IEEE Trans. Med. Imaging 35, 819–829 (2016). ITMID4 0278-0062 10.1109/TMI.2015.2497285 26552080 PMC4873963

[r22] Blacher S. et al., “Quantitative assessment of mouse mammary gland morphology using automated digital image processing and TEB detection,” Endocrinology 157, 1709–1716 (2016). ENDOAO 0013-7227 10.1210/en.2015-1601 26910307

[r23] Aeffner F. et al., “Quantitative assessment of pancreatic cancer precursor lesions in IHC-stained tissue with a tissue image analysis platform,” Lab Invest. 96, 1327–1336 (2016). 10.1038/labinvest.2016.111 27775692

[r24] Aeffner F. et al., “Validation of a muscle-specific tissue image-analysis tool for quantitative assessment of dystrophin staining in frozen muscle biopsies,” Arch. Pathol. Lab Med. 143(2), 197–205 (2018). 10.5858/arpa.2017-0536-OA 30168727 PMC6813837

[r25] Chen J. et al., “Computer-aided prognosis on breast cancer with hematoxylin and eosin histopathology images: a review,” Tumour Biol. 39, 1010428317694550 (2017). TUMBEA 10.1177/1010428317694550 28347240

[r26] Li X. et al., “Deep learning segmentation of glomeruli on kidney donor frozen sections,” J. Med. Imaging 8, 067501 (2021). JMEIET 0920-5497 10.1117/1.JMI.8.6.067501 PMC8685284 34950750

[27] Leo P. et al., “Computationally derived cribriform area index from prostate cancer hematoxylin and eosin images is associated with biochemical recurrence following radical prostatectomy and is most prognostic in Gleason grade group 2,” Eur. Urol. Focus 7(4), 722–732 (2021). 10.1016/j.euf.2021.04.016 33941504 PMC8419103

[28] Aeffner F. et al., “Introduction to digital image analysis in whole-slide imaging: a white paper from the digital pathology association,” J. Pathol. Inf. 10, 9 (2019). 10.4103/jpi.jpi_82_18 PMC6437786 30984469

[29] Aeffner F. et al., “Commentary: roles for pathologists in a high-throughput image analysis team,” Toxicol. Pathol. 44, 825–834 (2016). TOPADD 0192-6233 10.1177/0192623316653492 27343178

[30] Taqi S. A. et al., “A review of artifacts in histopathology,” J. Oral Maxillofac. Pathol. 22(2), 279 (2018). 10.4103/jomfp.JOMFP_125_15 PMC6097380 30158787

[31] Gadegbeku C. A. et al., “Design of the Nephrotic Syndrome Study Network (NEPTUNE) to evaluate primary glomerular nephropathy by a multidisciplinary approach,” Kidney Int. 83(4), 749–756 (2013). 10.1038/ki.2012.428 23325076 PMC3612359

[32] Mariani L. H. et al., “CureGN study rationale, design, and methods: Establishing a large prospective observational study of glomerular disease,” Amer. J. Kidney Dis. 73(2), 218–229 (2019). 10.1053/j.ajkd.2018.07.020 30420158 PMC6348011

[33] Bankhead P. et al., “Qupath: open source software for digital pathology image analysis,” Sci. Rep. 7(1), 16878 (2017). SRCEC3 2045-2322 10.1038/s41598-017-17204-5 29203879 PMC5715110

[34] Janowczyk A. Madabhushi A., “Deep learning for digital pathology image analysis: a comprehensive tutorial with selected use cases,” J. Pathol. Inf. 7, 29 (2016). 10.4103/2153-3539.186902 PMC4977982 27563488

[r35] Abels E. et al., “Computational pathology definitions, best practices, and recommendations for regulatory guidance: a white paper from the digital pathology association,” J. Pathol. 249(3), 286–294 (2019). 10.1002/path.5331 31355445 PMC6852275

[r36] LeCun Y. Bengio Y. Hinton G., “Deep learning,” Nature 521(7553), 436–444 (2015). 10.1038/nature14539 26017442

[37] Litjens G. et al., “A survey on deep learning in medical image analysis,” Med. Image Anal. 42, 60–88 (2017). 10.1016/j.media.2017.07.005 28778026

[38] Lafata K. J. et al., “Data clustering based on Langevin annealing with a self-consistent potential,” Q. Appl. Math. 77(3), 591–613 (2019). 10.1090/QAM/1521

[39] Stevens J. B et al., “Radiomics on spatial-temporal manifolds via Fokker-Planck dynamics,” Med. Phys. 51(5), 3334–3347 (2024). 10.1002/mp.16905 38190505

[40] Ji H. et al., “Post-radiotherapy PET image outcome prediction by deep learning under biological model guidance: A feasibility study of oropharyngeal cancer application,” Front. Oncol. 12, 895544 (2022). 10.3389/fonc.2022.895544 35646643 PMC9135979

[41] Yang Z. et al., “A neural ordinary differential equation model for visualizing deep neural network behaviors in multi-parametric MRI-based glioma segmentation,” Med. Phys. 50(8), 4825–4838 (2023). 10.1002/mp.16286 36840621 PMC10440249

