# Supplementary material for: Characterization of arteriosclerosis based on computer-aided measurements of intra-arterial thickness
Source: J Med Imaging (Bellingham). 2024 Oct 10;11(5):057501. doi: 10.1117/1.JMI.11.5.057501 (PMC11466048; doi:10.1117/1.JMI.11.5.057501)
Supplement: Supplementary file 1 [file JMI_011_057501_SD001.pdf]

Consortia Information and Acknowledgments

Members of the Nephrotic Syndrome Study Network (NEPTUNE)

**Principal Investigator; **Co-investigator; #Study Coordinator; §Providence Medical Research Center, Spokane, WA*

NEPTUNE Collaborating Sites

Atrium Health Levine Children's Hospital, Charlotte, SC: Susan Massengill[*], Layla Lo[#]
Cleveland Clinic, Cleveland, OH: Katherine Dell[*], John O'Toole[*], John Sedor[**], Blair Martin[#]
Children's Hospital, Los Angeles, CA: Ian Macumber[*], Silpa Sharma[#]
Children's Mercy Hospital, Kansas City, MO: Tarak Srivastava[*], Kelsey Markus[#]
Cohen Children's Hospital, New Hyde Park, NY: Christine Sethna[*], Suzanne Vento[#]
Columbia University, New York, NY: Pietro Canetta[*]
Duke University Medical Center, Durham, NC: Opeyemi Olabisi[*], Rasheed Gbadegesin[**], Maurice Smith[#]
Emory University, Atlanta, GA: Laurence Greenbaum[*], Chia-shi Wang[*], Emily Yun[#]
The Lundquist Institute, Torrance, CA: Sharon Adler[*], Janine LaPage[#]
John H Stroger Cook County Hospital, Chicago, IL: Amatur Amarah[*], Mathew Itteera[#]
Johns Hopkins Medicine, Baltimore, MD: Meredith Atkinson[*], Miahje Williams[#]
Mayo Clinic, Rochester, MN: John Lieske, Marie Hogan, Fernando Fervenza
Medical University of South Carolina, Charleston, SC: David Selewski[*], Cheryl Alston[#]
Montefiore Medical Center, Bronx, NY: Kim Reidy[*], Michael Ross[*], Frederick Kaskel[**], Patricia Flynn[#]
New York University Medical Center, New York, NY: Laura Malaga-Dieguez[*], Olga Zhdanova[**], Laura Jane Pehrson[#], Melanie Miranda[#]
The Ohio State University College of Medicine, Columbus, OH: Salem Almaani[*], Laci Roberts[#]
Stanford University, Stanford, CA: Richard Lafayette[*], Shiktij Dave[#]
Temple University, Philadelphia, PA: Iris Lee[**]
Texas Children's Hospital at Baylor College of Medicine, Houston, TX: Shweta Shah[*], Sadaf Batla[# #]
University Health Network Toronto: Heather Reich[*], Michelle Hladunewich[**], Paul Ling[#], Martin Romano[#]
University of California at San Francisco, San Francisco, CA: Paul Brakeman[*]
University of Colorado Anschutz Medical Campus, Aurora, CO: James Dylewski[*] Nathan Rogers[#]
University of Kansas Medical Center, Kansas City, KS: Ellen McCarthy[*], Catherine Creed[#]
University of Miami, Miami, FL: Alessia Fornoni[*], Miguel Bandes[#]
University of Michigan, Ann Arbor, MI: Matthias Kretzler[*], Laura Mariani[*], Zubin Modi[*], A Williams[#], Roxy Ni[#]
University of Minnesota, Minneapolis, MN: Patrick Nachman[*], Michelle Rheault[*], Amy Kowalski[#], Nicolas Rauwolf[#]
University of North Carolina, Chapel Hill, NC: Vimal Derebail[*], Keisha Gibson[*], Anne Froment[#], Sara Kelley[#]
University of Pennsylvania, Philadelphia, PA: Lawrence Holzman[*], Kevin Meyers[**], Krishna Kallem[#], Aliya Edwards[#]
University of Texas San Antonio, San Antonio, TX: Samin Sharma[**]
University of Texas Southwestern, Dallas, TX: Elizabeth Roehm[*], Kamalanathan Sambandam[**], Elizabeth Brown[**], Jamie Hellewege
University of Washington, Seattle, WA: Ashley Jefferson[*], Sangeeta Hingorani[**], Katherine Tuttle[**§], Linda Manahan[#], Emily Pao[#], Kelli Kuykendall[§]
Wake Forest University Baptist Health, Winston-Salem, NC: Jen Jar Lin[**]
Washington University in St. Louis, St. Louis, MO: Vikas Dharnidharka[*]

Data Analysis and Coordinating Center: *University of Michigan:* Matthias Kretzler[*], Brenda Gillespie[**], Laura Mariani[**], Zubin Modi[**], Eloise Salmon[**], Howard Trachtman[**], Tina Mainieri, Gabrielle Alter, Michael Arbit, Hailey Desmond, Sean Eddy, Damian Fermin, Wenjun Ju, Maria Larkina, Chrysta Lienczewski, Rebecca Scherr, Jonathan Troost, Amanda Williams, Yan Zhai; *Arbor Collaborative for Health:* Colleen Kincaid, Shengqian Li, Shannon Li; *Cleveland Clinic:* Crystal Gadegbeku[**], *Duke University:* Laura Barisoni[**]; John Sedor[**], *Harvard University:* Matthew G Sampson[**]; *Northwestern University:* Abigail Smith[**]; *University of Pennsylvania:* Lawrence Holzman[**], Jarcy Zee[**]

Digital Pathology Committee: Carmen Avila-Casado *(University Health Network)*, Serena Bagnasco *(Johns Hopkins University)*, Lihong Bu *(Mayo Clinic)*, Shelley Caltharp *(Emory University)*, Clarissa Cassol *(Arkana)*, Dawit Demeke *(University of Michigan)*, Brenda Gillespie *(University of Michigan)*, Jared Hassler *(Temple University)*, Leal Herlitz *(Cleveland Clinic)*, Stephen Hewitt *(National Cancer Institute)*, Jeff Hodgin *(University of Michigan)*, Danni Holanda *(Arkana)*, Neeraja Kambham *(Stanford University)*, Kevin Lemley, Laura Mariani *(University of Michigan)*, Nidia Messias *(Washington University)*, Alexei Mikhailov *(Wake Forest)*, Vanessa Moreno *(University of North Carolina)*, Behzad Najafian *(University of Washington)*, Matthew Palmer *(University of Pennsylvania)*, Avi Rosenberg *(Johns Hopkins University)*, Virginie Royal *(University of Montreal)*, Miroslav Sekulik *(Columbia University)*, Barry Stokes *(Columbia University)*, David Thomas *(Duke University)*, Ming Wu *(University of New York)*, Michifumi Yamashita *(Cedar Sinai)*, Hong Yin *(Emory University)*, Jarcy Zee *(University of Pennsylvania)*, Yiqin Zuo *(University of Miami)*. Co-Chairs: Laura Barisoni *(Duke University)*, Cynthia Nast *(Cedar Sinai)*.

CureGN Collaborators

The CureGN Consortium members listed below, from within the four Participating Clinical Center networks and Data Coordinating Center, are acknowledged by the authors as Collaborators.
**CureGN Principal Investigators; *CureGN Site Principal Investigators; [#]CureGN Lead Coordinators.

CureGN Participating Clinical Centers (PCC) through Columbia University:
Columbia University, New York, NY, US: Wooin Ahn, Gerald Appel, Paul Appelbaum, Revekka Babayev, Andrew Bomback, Pietro Canetta, Brenda Chan, Vivette Denise D'Agati, Samitri Dogra, Hilda Fernandez, Ali Gharavi**, William Hines, Syed Ali Husain, Namrata Jain, Krzysztof Kiryluk, Fangming Lin, Maddalena Marasa[#], Glen Markowitz, Hila Milo Rasouly, Sumit Mohan, Nicola Mongera, Jordan Nestor, Thomas Nickolas, Jai Radhakrishnan, Maya Rao, Simone Sanna-Cherchi, Shayan Shirazian, Michael Barry Stokes, Natalie Uy, Anthony Valeri, Natalie Vena
University of Warsaw, Warszawa, Poland: Bartosz Foroncewicz, Barbara Moszczuk, Krzysztof Mucha*, Agnieszka Perkowska-Ptasińska
Gaslini Children's Hospital, Genoa, Italy: Gian Marco Ghiggeri*, Francesca Lugani

CureGN Participating Clinical Centers (PCC) through the Pediatric Nephrology Research Consortium:
Arkana Laboratories, Little Rock, AR, USA: Josephine Ambruzs, Helen Liapis
Children's Hospital of Michigan, Detroit, MI, USA: Rossana Baracco, Amrish Jain*
Children's Hospital of New Orleans/ LSU Health, New Orleans, LA, USA: Isa Ashoor, Diego Aviles*
Children's Mercy Hospital, Kansas City, MO, USA: Tarak Srivastava*
Children's National Medical Center, Washington DC, USA: Sun-Young Ahn*
Cincinnati Children's Hospital Cincinnati, OH, USA: Prasad Devarajan, Elif Erkan*, Donna Claes, Hillarey Stone
Connecticut Children's Medical Center, Hartford, CT, USA: Sherene Mason*
Duke Children's Hospital Medical Center, Durham, NC, USA: Rasheed Gbadegesin*
East Carolina University Brody School of Medicine, Greenville, NC, USA: Liliana Gomez-Mendez*
Emory University, Atlanta, GA, USA: Larry Greenbaum**, Chia-shi Wang, Hong (Julie) Yin
Helen DeVos Children's Hospital, Grand Rapids, MI, USA: Yi Cai*, Goebel Jens, Julia Steinke
Levine Children's Hospital/Atrium Health, Charlotte, NC, USA: Donald Weaver*
Lurie Children's Hospital, Chicago IL, USA: Jerome Lane*
Mayo Clinic, Rochester, MN, USA: Carl Cramer*
Medical College of Wisconsin, Milwaukee, WI, USA: Cindy Pan, Neil Paloian, Rajasree Sreedharan*
Medical University of South Carolina, Charleston SC, USA: David Selewski, Katherine Twombley*
Nationwide Children's Hospital, Columbus, OH, USA: Corinna Bowers[#], Mary Dreher[#] Mahmoud Kallash*, John Mahan, Samantha Sharpe[#], William Smoyer**
Oregon Health and Science University, Portland, OR, USA: Amira Al-Uzri*, Sandra Iragorri
Riley Children's Hospital, Indianapolis, IN, USA: Myda Khalid*
Cardinal Glennon Children's Medical Center/ St. Louis University, St. Louis, MO, USA: Craig Belsha*
Texas Children's Hospital, Houston, TX, USA: Joseph Alge*, Michael Braun, AC Gomez, Scott Wenderfer*
Texas Tech Health Sciences Center, Amarillo, TX, USA: Tetyana Vasylyeva*
Children's of Alabama, University of Alabama, Birmingham, AL, USA: Daniel Feig*

University of Colorado Children's Hospital, Colorado, Aurora, CO, USA: Gabriel Cara Fuentes, Melisha Hannah*
University of Iowa Children's Hospital, Iowa City, IA, USA: Carla Nester*
University of Kentucky, Lexington, KY, USA: Aftab Chishti*
University of Louisville, Louisville, KY, USA: Jon Klein**
Holtz Medical Center, University of Miami, Miami, FL, USA: Chryso Katsoufis, Wacharee Seeherunvong*
University of Minnesota Children's Hospital, Minneapolis, MN, USA: Michelle Rheault*
University of New Mexico Health Sciences Center, Albuquerque, NM, USA: Craig Wong*
University of Oklahoma Health Sciences Center, Oklahoma City, OK, USA: Nisha Mathews*
University of Virginia, Charlottesville, VA, USA: John Barcia*, Agnes Swiatecka-Urban
University of Wisconsin, Madison, WI, USA: Sharon Bartosh*
Vanderbilt Children's Hospital, Nashville TN, USA: Tracy Hunley*
Washington University in St. Louis, St. Louis, MO, USA: Vikas Dharnidharka*, Joseph, Gaut

CureGN Participating Clinical Centers (PCC) through the University of North Carolina:
Hôpital Maisonneuve-Rosemont, Montreal, Canada: Louis-Philippe Laurin*, Virginie Royal
Medical University of South Carolina, Charleston, SC, USA: Anand Achanti, Milos Budisavljevic*, Sally Self
Northwestern University, Chicago, IL, USA: Cybele Ghossein, Yonatan Peleg, Shikha Wadhwani*
Ohio State University, Columbus, OH, USA: Salem Almaani, Isabelle Ayoub, Tibor Nadasdy, Samir, Parikh, Brad Rovin*
University of Chicago, Chicago, IL, USA: Anthony Chang
University of Alabama at Birmingham, Birmingham, AL, USA: Huma Fatima, Bruce Julian, Jan Novak, Matthew Renfrow, Dana Rizk*
University of North Carolina Kidney Center, Chapel Hill, NC, USA: Dhruti Chen, Vimal Derebail, Ronald Falk**, Keisha Gibson, Dorey Glenn, Susan Hogan, Koyal Jain, J. Charles Jennette, Amy Mottl*, Caroline Poulton#, Manish Kanti Saha
Vanderbilt University, Nashville, TN, USA: Agnes Fogo, Neil Sanghani*
Virginia Commonwealth University, Richmond, VA, USA: Jason Kidd*, Selvaraj Muthusamy

CureGN Participating Clinical Centers (PCC) through the University of Pennsylvania:
MetroHealth Medical Center/Case Western Reserve University, Cleveland, OH, USA: Jeffrey Schelling*
Cedars-Sinai Health System, Los Angeles, CA, USA: Jean Hou
Children's Hospital of LA, Los Angeles, CA, USA: Kevin Lemley*, Warren Mika, Pierre Russo
Children's Hospital of Philadelphia, Philadelphia, PA, USA: Michelle Denburg, Amy Kogon, Kevin Meyers*, Madhura Pradhan
Cleveland Clinic, Cleveland, OH, CA: Raed Bou Matar*, John O'Toole*, John Sedor*
Cohen Children's Medical Center, New Hyde Park, NY, USA: Christine Sethna*, Suzanne Vento #
Johns Hopkins University, Baltimore, MD, USA: Mohamed Atta, Serena Bagnasco, Alicia Neu, John Sperati*
Lundquist Institute at Harbor-UCLA Medical Center, Torrance, CA, USA: Sharon Adler*, Tiane Dai, Ram Dukkipati
Mayo Clinic, Rochester, MN, USA: Fernando Fervenza*, Sanjeev Sethi
Montefiore Medical Center, The Bronx, New York, NY, USA: Frederick Kaskel, Kaye Brathwaite, Kimberly Reidy*
New York University, New York, NY, USA: Joseph Weisstuch, Ming Wu, Olga Zhdanova
NIDDK, Bethesda, MD, USA: Jurgen Heymann, Jeffrey Kopp*, Meryl Waldman, Cheryl Winkler
Spokane Providence Medical Center, Spokane, WA, USA: Katherine Tuttle*
Stanford University, Palo Alto, CA, USA: Jill Krissberg, Richard Lafayette*, Kamal Fahmeedah, Elizabeth Talley
Sunnybrook Health Sciences Centre, Toronto, Canada: Michelle Hladunewich*
The Hospital for Sick Children, Toronto, Canada: Rulan Parekh*
University Health Network, Toronto, Canada: Carmen Avila-Casado, Daniel Cattran*, Reich Heather, Philip Boll
University of Miami, Miami, FL, USA: Yelena Drexler, Alessia Fornoni*
University of Michigan, Ann Arbor, MI, USA: Brooke Blazius*, Jeffrey Hodgin, Andrea Oliverio
University of Pennsylvania, Philadelphia, PA, USA: Jon Hogan, Lawrence Holzman**, Matthew Palmer, Gaia Coppock
University of Pittsburgh School of Medicine, Pittsburgh, PA, USA: Blaise Abromovitz*, Michael Mortiz*
University of Washington, Seattle, WA, USA: Charles Alpers, J. Ashley Jefferson*
UT Southwestern, Dallas, TX, USA: Elizabeth Brown, Kamal Sambandam*, Bethany Roehm

Data Coordinating Center (DCC):
Arbor Research Collaborative for Health, Ann Arbor, MI, USA: John Graff, Abigail Smith
Cedar Sinai Medical Center, Los Angeles, CA, USA: Cynthia Nast
Duke University, Durham, NC, USA: Laura Barisoni
University of Michigan, Ann Arbor, MI, USA: Brenda Gillespie**, Bruce Robinson**, Matthias Kretzler, Laura Mariani**

Steering Committee Chair: Lisa M. Guay-Woodford, Children's Hospital of Pennsylvania, Philadelphia, PA, USA
